# Single-cell transcriptome analyses reveal distinct gene expression signatures of severe COVID-19 in the presence of clonal hematopoiesis

**DOI:** 10.1038/s12276-022-00866-1

**Published:** 2022-10-13

**Authors:** Baekgyu Choi, Chang Kyung Kang, Seongwan Park, Dohoon Lee, Andrew J. Lee, Yuji Ko, Suk-Jo Kang, Kyuho Kang, Sun Kim, Youngil Koh, Inkyung Jung

**Affiliations:** 1 Department of Biological Sciences, Korea Advanced Institute of Science and Technology (KAIST), Daejeon, 34141 Republic of Korea; 2 Department of Internal Medicine, Seoul National University College of Medicine, Seoul, 03080 Republic of Korea; 3 Bioinformatics Institute, Seoul National University, Seoul, 08826 Republic of Korea; 4 Department of Biology, Chungbuk National University, Cheongju, 28644 Republic of Korea; 5 Department of Computer Science and Engineering, College of Engineering, Seoul National University, Seoul, 08826 Republic of Korea; 6 Interdisciplinary Program in Bioinformatics, College of Natural Sciences, Seoul National University, Seoul, 08826 Republic of Korea; 7 Genome Opinion Inc, Seoul, 04799 Republic of Korea

**Keywords:** Epigenetics in immune cells, Viral infection

## Abstract

Clonal hematopoiesis of indeterminate potential (CHIP), a common aging-related process that predisposes individuals to various inflammatory responses, has been reported to be associated with COVID-19 severity. However, the immunological signature and the exact gene expression program by which the presence of CHIP exerts its clinical impact on COVID-19 remain to be elucidated. In this study, we generated a single-cell transcriptome landscape of severe COVID-19 according to the presence of CHIP using peripheral blood mononuclear cells. Patients with CHIP exhibited a potent IFN-γ response in exacerbating inflammation, particularly in classical monocytes, compared to patients without CHIP. To dissect the regulatory mechanism of CHIP (+)-specific IFN-γ response gene expression in severe COVID-19, we identified *DNMT3A* CHIP mutation-dependent differentially methylated regions (DMRs) and annotated their putative target genes based on long-range chromatin interactions. We revealed that CHIP mutant-driven hypo-DMRs at poised *cis*-regulatory elements appear to facilitate the CHIP (+)-specific IFN-γ-mediated inflammatory immune response. Our results highlight that the presence of CHIP may increase the susceptibility to hyperinflammation through the reorganization of chromatin architecture, establishing a novel subgroup of severe COVID-19 patients.

## COVID-19: Specific subgroup susceptible to severe disease

An aging-related blood condition can trigger hyperinflammation across the body during COVID-19, enhancing the risk of severe disease. Clonal hematopoiesis of indeterminate potential (CHIP) occurs when stem cells begin making blood cells with a specific mutation, leading to groups of cells with a different genetic signature than the rest of the blood cells. CHIP can predispose people to particular inflammatory responses and even cancers. Inkyung Jung at the Korea Advanced Institute of Science and Technology, Daejeon, and co-workers demonstrated how CHIP can make people more susceptible to severe COVID-19 infection. The team analyzed samples from COVID-19 patients with and without CHIP at single-cell resolution. In those with CHIP, they identified a strong proinflammatory response in certain circulating white blood cells, which exacerbated systemic inflammation. Anti-inflammatory therapies that target these reponses may provide possible treatments.

## Introduction

Coronavirus disease-19 (COVID-19), an emerging infectious disease caused by severe acute respiratory syndrome-coronavirus-2 (SARS-CoV-2) infection, has become the global health threat of the century^1^. Epidemiological data have revealed that approximately 20% of individuals with COVID-19 have a severe or critical illness course^2^. Many clinical risk factors for severe COVID-19, including older age, comorbidities such as diabetes mellitus or hypertension, and morbid obesity, have been found^3,4^.

Clinical deterioration, such as acute respiratory distress syndrome or intensive care unit admission, most commonly occurs around the 10th day of illness^5,6^, when viral loads decline after the early peak^7,8^. This temporal discrepancy suggests that immunological phenomena may play an important role in the clinical manifestations of COVID-19. High levels of circulating proinflammatory cytokines^9^, aberrant hyperactivation of cytotoxic lymphocytes^10^ and their infiltration in vital organs^11^, and dysregulated monocytes and macrophages^12^ have each been proposed as mechanisms for the pathological immune responses in severe COVID-19. However, identifying additional factors driving the severity of severe COVID-19 remains a challenge.

Recently, several studies have highlighted the clinical impact of clonal hematopoiesis of indeterminate potential (CHIP) on COVID-19 severity^13–15^. CHIP refers to a population of immune cells with acquired gene mutations that do not fulfill the diagnostic criteria for hematological malignancy^16^. As the majority of genes associated with CHIP, including *DNMT3A*, *TET2*, and *ASXL1*, are involved in epigenetic regulation, CHIP may have a wide range of effects on immune function through altered chromatin activities^17^. There is growing evidence supporting the role of CHIP mutations in altered immune function through effector cells such as monocytes/macrophages and their dysregulated cytokine/chemokine expression^18–21^, which largely shares the immunopathogenic signatures of severe COVID-19. In this regard, a recent examination of over 500 COVID-19 patients revealed a statistical association between the presence of CHIP and COVID-19 severity^13^. However, the CHIP-specific immune responses and the exact gene expression program on how the presence of CHIP exerts its clinical impact on the progression of severe COVID-19 are not clear^13–15^.

To address this issue, we aimed to explore the CHIP-dependent gene expression program in severe COVID-19 by using single-cell immune transcriptome landscapes of normal controls, mild COVID-19 patients, and severe COVID-19 patients according to the presence of CHIP. We also investigated how CHIP contributes to the immunological responses in severe COVID-19 through CHIP-dependent dysregulated epigenetic gene regulation mechanisms.

## Materials and methods

For more detailed protocols, see the Supplementary Methods.

### Single-cell RNA-seq experiments on CHIP (+) specimens

A total of 10 CHIP (+) specimens were newly collected and confirmed by custom designed probes (Agilent, Santa Clara, CA) targeting known CHIP variants to generate single-cell transcriptome profiles. Two CHIP (+) SARS-CoV-2-uninfected normal specimens were obtained from patients with a history of plasma cell dyscrasia in remission. CHIP (+) severe (*n* = 4) and CHIP (+) mild (*n* = 4) COVID-19 specimens were collected from laboratory-confirmed COVID-19 patients between February and June 2020 at Seoul National University Hospital in the Republic of Korea (IRB Nos. 2003-141-1110). For the use of patient samples, an expedited review was performed by the Institutional Review Board (IRB) committee of the Korea Advanced Institute of Science and Technology, and an IRB review exemption was obtained (IRB Nos. IRB-21-269). Peripheral blood mononuclear cells (PBMCs) were isolated from peripheral venous blood via standard Ficoll-Paque (GE Healthcare, Uppsala, Sweden) density gradient centrifugation, frozen in freezing media, and stored in liquid nitrogen until use. After thawing the samples, the cells were washed twice with chilled PBS containing 0.04% BSA and filtered through a Flowmi Tip Strainer (40 μm, Bel-Art SP Scienceware, Wayne, NJ, USA). Then, we performed single-cell RNA-seq (scRNA-seq) experiments using the Chromium Single Cell 3′ Library & Gel Bead Kit v3 (10x Genomics, Pleasanton, CA) following the manufacturer’s instructions. The constructed libraries were sequenced at a depth of over 50,000 reads per cell using DNBSEQ-G400 (MGI, Shenzhen, China), except for CHIP (+) mild COVID-19, which were sequenced with a depth of over 20,000 reads per cell. The list of samples and sequencing results are summarized in Supplementary Table 1.

### Single-cell RNA-seq data processing

The fastq files from outputs of DNBSEQ-G400 were demultiplexed into each sample using splitBarcode (https://github.com/MGI-tech-bioinformatics/splitBarcode, v0.1.6). The raw data from the previous study were downloaded from the Gene Expression Omnibus (GEO) database under accession number GSE149689. Among the public datasets, we confirmed the existence of mutations in the mild and severe COVID-19 patients, finding one patient in the severe group who had CHIP mutations (Supplementary Tables 2, 3). The list of all single-cell RNA-seq samples is provided in Supplementary Tables 1 and 3. We aligned the demultiplexed reads to the human reference genome (GRCh38; 10x cellranger reference GRCh38 v3.0.0) using the cellranger count (v3.0.2)^22^. All aligned data were integrated by cellranger aggr (v3.0.2) using default parameters except for CHIP (+) mild COVID-19 patients and uninfected donors, which were separately aggregated with the same method. We used Seurat R package v3.1.5^23^ to perform the following analysis. After generating the feature-barcode matrix through cellranger, we excluded cells that expressed <200 genes or genes that were not expressed in any cells. For the scRNA-seq data of CHIP (+) mild COVID-19 and CHIP (+) uninfected donors, cells were classified into each individual type according to their genotype with the Souporcell package^24^, removing heterogeneous doublets between individuals. Moreover, we eliminated low-quality cells and doublets from our data according to the following criteria: cells with mitochondrial gene expression as >15% of their total gene expression and cells with <1000 and >15,000 unique molecular identifier (UMI) counts. For each cell, the raw gene expression counts were normalized by the total UMI count and log-transformed. To find features to merge the data from different experiments, we used the vst method in Seurat R package v3.2.0 and identified 2000 highly variable genes from each sample. Through canonical correlation analysis, the data were aligned using anchors based on the top 10 canonical correlation vectors. Then, we scaled the aligned data and conducted principal component analysis (PCA). Using the top 15 principal components (PCs), clustering was performed with a resolution parameter value of 0.8, and the cells were visualized by UMAP projection.

### Cell type annotation through marker gene identification in each cluster

To identify marker genes, upregulated genes in each cluster relative to the other clusters were selected based on the results of the Wilcoxon rank-sum test in Seurat’s implementation with >0.25 log fold change compared to the other clusters and a Bonferroni-adjusted *P* < 0.05. By manual inspection, among the 24 different clusters, 16 were assigned to 9 known immune cell types, red blood cells, and platelets. The clusters characterized by similar marker genes were manually combined into one cell type.

### Projection of scRNA-seq data to reference data

**Fig. 1:**
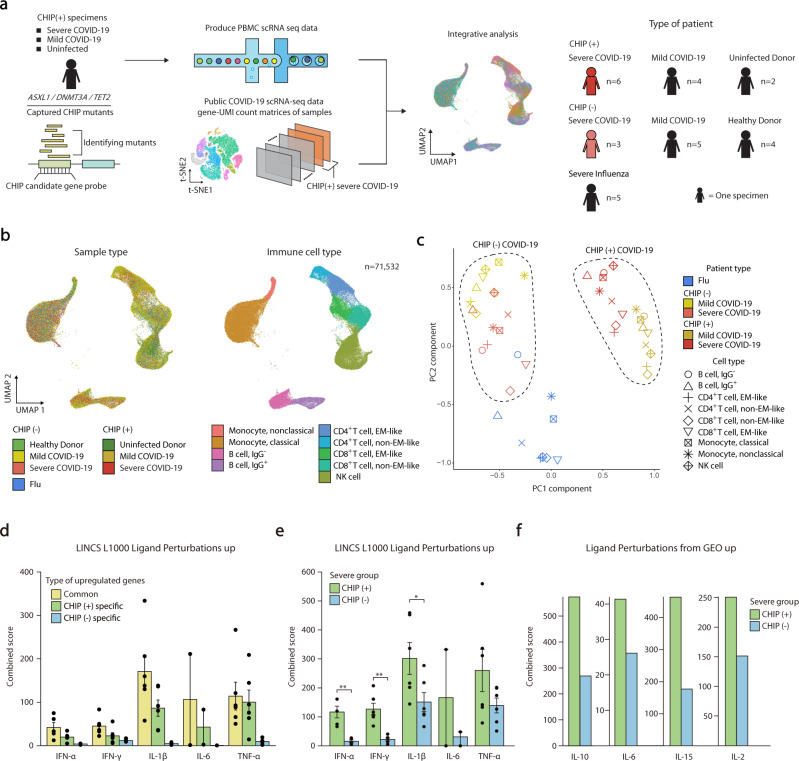
Single-cell transcriptome analyses of COVID-19 according to CHIP status. **a** Overview of the study design. **b** Scatter plots of integrated scRNA-seq data represented by the UMAP method. Left, colored by group information. Right, annotated on immune cells. **c** PCA of transcriptome profiles according to immune cell types and disease groups. **d** Bar plots showing combined scores of the LINCS L1000 Ligand Perturbations up gene ontology library for upregulated genes of severe CHIP (+) and CHIP (-) compared to CHIP (-) mild COVID-19. Each point indicates one ligand perturbation term in the library: IFN-α (*n* = 5), IL-1β (*n* = 6), IFN-γ (*n* = 6), IL-17 (*n* = 2), IL-6 (*n* = 2) and TNF-α (*n* = 6). The mean and standard error of the mean (SEM) of each gene set are shown. **e** Bar plots showing combined scores of DEGs in classical monocytes between CHIP (+) and CHIP (-) severe COVID-19 for the same gene ontology library in (**d**). The mean and standard error of the mean (SEM) of each gene set are shown. A one-sided Mann‒Whitney’s *U* test was performed (*P < 0.05, **P < 0.01). **f** Bar plots showing combined scores of the same gene sets in (**e**) for Ligand Perturbations from GEO up gene ontology library.

The single-cell transcriptome data from CHIP (+) uninfected donors and CHIP (+) mild COVID-19 patients were projected as a query to the rest of the data through the Seurat R package. To project the query onto the reference data, anchors between two datasets were calculated through the FindTransferAnchors function using the top 50 principal components from the reference data. The cell type of each cell in the query data was predicted based on those anchors through the TransferData function within 50 dimensions for the anchor weighting procedure. The query data were then projected to the reference data with MapQuery (reference.reduction as ‘pca’, reduction.model as ‘umap’), generating the whole UMAP plot in Fig. 1b.

### Identification of DEGs using MAST

We used the model-based analysis of single-cell transcriptomics (MAST, v1.16.0) algorithm^25^ in Seurat’s implementation to identify differentially expressed genes (DEGs) between the two groups. The significant DEGs were defined based on Bonferroni-adjusted *P* < 0.05 and a log2 fold change >0.25. Mitochondrial genes and ribosomal genes were excluded from the DEG analysis. One sample from an asymptomatic mild COVID-19 patient was excluded during the calculation of differentially expressed genes, similar to a previous study^26^.

### Enrichment analysis using enrichr

**Fig. 2:**
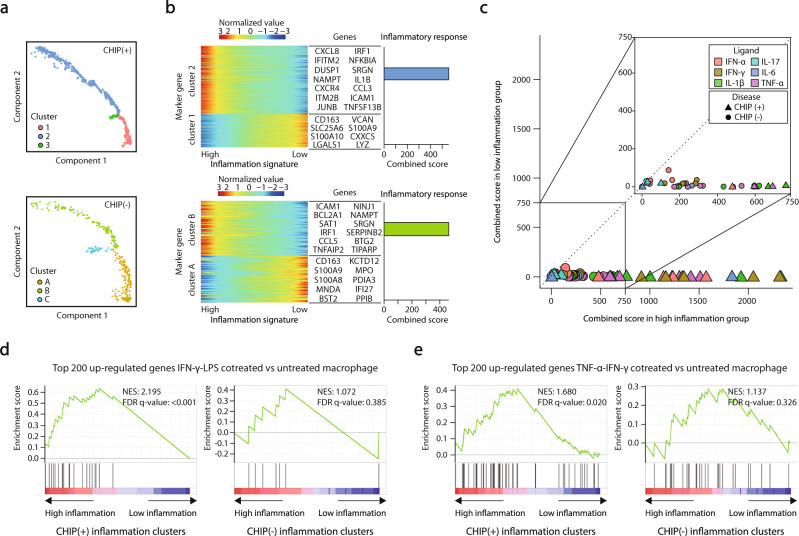
Pseudotime analyses of severe COVID-19 with CHIP in classical monocytes. **a**–**c** Pseudotime analyses for CHIP (+) or CHIP (-) severe COVID-19 patients. **a** Cells were aligned according to the pseudotime axis calculated using Monocle2. The color indicates the type of cluster. Top, CHIP (+) patients. Bottom, CHIP (-) patients. **b** Heatmaps representing the expression levels of marker genes for the ordered cells. The list of genes is the representative gene set for each cluster. Bar plots showing the combined scores of each cluster for the inflammatory response term in MsigDB Hallmark 2020. **c** Scatter plots of the combined scores of marker genes of inflammation clusters for the LINCS L1000 Ligand Perturbations up gene ontology library. Horizontal axis, high inflammation cluster; vertical axis, low inflammation cluster. The identity lines are presented diagonally. The shapes represent the type of patients. Colors indicate the types of perturbed ligands for IFN-α (*n* = 5), IL-1β (*n* = 6), IFN-γ (*n* = 6), IL-17 (*n* = 2), IL-6 (*n* = 2) and TNF-α (*n* = 6). **d**, **e** GSEA plots for marker genes of inflammation clusters in CHIP (+) and CHIP (-) individuals, respectively. Genes are ordered based on log-fold changes between high- and low-inflammation clusters. Normalized enrichment scores (NES) and FDR are presented for (**d**) DEGs between IFN-γ-LPS vs. untreated human macrophages^41^ and (**e**) DEGs between TNF-α-IFN-γ cotreatment and untreated conditions^39^.

All gene ontology libraries analyzed in this study were collected from the enrichR database, implemented by the R package ‘enrichR’ (v3.0)^27^. By entering the gene sets and name of the gene ontology library into the ‘enrichr’ function in the package, enriched terms in the library and enrichment scores of gene sets were returned. Scatter plots or bar plots were drawn with returned values. For enrichment scores, a combined score was used. The combined score was computed by taking the log of the *p* value from Fisher’s exact test and multiplying it by the z score of the deviation from the expected rank. The list of libraries used for the figures are as follows: MSigDB Hallmark 2020 (Fig. 2b), LINCS L1000 Ligand Perturbations up (Figs. 1d, e, 2c, 4c, and Supplementary Figs. 4, 8c, d), and Ligand Perturbations from GEO up (Fig. 1f).

### Trajectory analysis

Trajectory analysis was applied to two double-sampled severe COVID-19 patients using Monocle2 (v2.18.0) (Fig. 2a–c)^28^. The exact processing steps can be found in the Supplementary Methods. Using the MSigDB Hallmark 2020 gene ontology library, clusters showing high combined scores for an inflammatory response term were annotated as high inflammation clusters, and the rest were annotated as low inflammation clusters. The pseudotime trajectory was labeled as inflammation signature, and the direction where the expression of marker genes of high inflammation clusters increased was denoted as high whereas the other direction was denoted as low (Fig. 2b).

### Subclustering analysis

The subclustering analysis was performed using classical monocytes from all CHIP (+) individuals by the Seurat R package. The expression levels of the top 1000 highly variable genes, selected based on vst, were scaled by ScaleData in Seurat, while individual variances were regressed out using the vars.to.regress option. Following computing the 10 principal components by RunPCA, the cells were clustered and visualized using FindNeighbors, FindClusters (0.2 resolution), and RunUMAP.

### Signature score calculation

To compute the score of multiple gene expressions in each cell, we utilized a software called Single-Cell Signature Scorer^29^. Briefly, the normalized expression matrix was used to calculate the signature score. The score of the given gene set in each cell was computed as a sum of UMI counts for all genes from the gene set in a cell divided by the total UMI counts in the cell.

### Monocyte sample preparation and in situ Hi-C experiment

For the use of human samples, Institutional Review Board (IRB) approval was obtained from KAIST (KH2017-63). Whole blood was obtained from one male and one healthy female volunteer of Korean ethnicity. Both donors were required to fast overnight to reduce dietary effects. Peripheral blood mononuclear cells (PBMCs) were obtained from whole blood by density gradient centrifugation (Ficoll-Paque), and then, two rounds of monocyte isolation (Miltenyi Biotec’s MACS system, #130-091-153) were performed to purify classical monocytes. In situ Hi-C was performed using a previously published protocol^30^. Libraries were sequenced on a HiSeq 4000 (Illumina, San Diego, USA).

### Analysis of histone ChIP-seq results in classical monocytes

To annotate the regulatory roles of differentially methylated regions (DMRs), ChIP-seq data for each histone modification were collected from the ENCODE portal (https://www.encodeproject.org/)^31^. Three distinct types of data, the fold change over the control bigwig file, signal *p* value bigwig file and narrow peak bed file, were downloaded (Figs. 4, 5 and Supplementary Fig. 8). Each profile and heatmap were drawn by the plot profile and plotHeatmap functions in Deeptools (v3.5.1), respectively. Narrow peak bed files were downloaded for the annotation of chromatin states (Fig. 5e and Supplementary Fig. 8e).

To observe the enrichment patterns of active signals in DMRs when inflammation occurs, two different replicates of ChIP-seq data for H3K27ac peaks of IFN-γ-LPS-treated human macrophages were downloaded from the GEO database (GSM1057019, replicate 1; GSM1057023, replicate 2)^32^. Genomic coordinates of downloaded data were converted from hg19 to hg38 using CrossMap (v0.5.2)^33^ with the hg19tohg38 chain file provided in the UCSC genome browser. The number of intersections between hypo-DMRs and H3K27ac peaks was used to perform Fisher’s exact test.

### Annotation of chromatin states for hypo-DMRs

We first classified hypo-DMRs into promoter-proximal and distal hypo-DMRs. Hypo-DMRs overlapping at least one base with 1.5 kb upstream and downstream of the transcription start site of protein-coding genes annotated by GENCODE (v.28) were considered promoter-proximal hypo-DMRs. The remaining ones were annotated as promoter-distal hypo-DMRs. Hypo-DMRs were also annotated with four different chromatin states defined by the combination of intersected histone modification peaks: for promoter-proximal hypo-DMRs, Active—H3K4me3 (+) and H3K27me3 (-); Bivalent—H3K4me3 (+) and H3K27me3 (+); Inactive—H3K4me3 (-) and H3K27me3 (+); and Others—H3K4me3 (-) and H3K27me3 (-); for promoter-distal hypo-DMRs, Active—H3K27ac (+) and H3K27me3 (-); Poised—H3K4me1 (+), H3K27ac (-) and H3K27me3 (-); Inactive—H3K27ac (-) and H3K27me3 (+); and others—H3K4me1 (-), H3K27ac (-) and H3K27me3 (-). (+) means intersection occurs between hypo-DMR and merged peak data and (-) means intersection does not occur.

### Statistical analyses

Student’s *t* test or Mann‒Whitney’s *U* test was used to compare continuous variables, while the chi-squared test or Fisher’s exact test was used to compare categorical variables. The Kolmogorov‒Smirnov test was used to compare two groups without the assumption of normality. Each statistical test was applied according to data size and distribution. The sample size was chosen by the availability of CHIP (+) samples for scRNA-seq experiments. We produced ten CHIP (+) scRNA-seq datasets and found two additional CHIP (+) samples from a previous study.

## Results

### Single-cell gene expression profiling of CHIP (+) severe COVID-19 demonstrates distinct immunological signatures

To understand the immunological signature of CHIP (+) severe COVID-19, we examined the single-cell immunological signatures of severe COVID-19 according to the presence of CHIP (Fig. 1a). We integrated a total of 79,011 high-quality single-cell transcriptome profiles of peripheral blood mononuclear cells (PBMCs) generated by the 10x Genomics single-cell RNA-seq (scRNA-seq) platform, comprising healthy control (*n* = 4), CHIP (+) uninfected control (*n* = 2), severe influenza (*n* = 5), CHIP (-) mild COVID-19 (*n* = 5), CHIP (-) severe COVID-19 (*n* = 3), CHIP (+) mild COVID-19 (*n* = 4), and CHIP (+) severe COVID-19 (*n* = 6) specimens (Supplementary Tables 1, 3, 4, see Methods). The reproducibility and quality were ensured (Supplementary Fig. 1)^26^. Based on uniform manifold approximation and projection (UMAP) of transcriptome profiles, 9 major immune cell types were assigned with previously annotated marker genes (Fig. 1b and Supplementary Fig. 2, see Methods)^26^.

In terms of normalized transcriptome profiles against healthy controls at the cell type resolution, as expected, all immune cell types originating from COVID-19 were clustered together when compared to influenza (Fig. 1c). Interestingly, patients with COVID-19 were subdivided according to the presence of CHIP (Fig. 1c). To identify the factors driving CHIP-dependent separation of COVID-19 severity, we identified the differentially expressed genes (DEGs) of CHIP (+) and CHIP (-) severe COVID-19 compared to CHIP (-) mild COVID-19 (see Methods). We annotated these genes into commonly (*n* = 278), CHIP (+)-specific (*n* = 807), and CHIP (-)-specific (*n* = 270) upregulated genes (Supplementary Table 5). Based on cytokine-responsive gene sets (see Methods)^34^, we found that the commonly upregulated genes were enriched by strong IL-1β and TNF-α responses (Fig. 1d). The commonly and CHIP (-)-specific upregulated genes were also significantly enriched by the genes associated with a type I IFN-mediated TNF/IL-1β-driven hyperinflammation immune signature, as we proposed in our previous study (Supplementary Fig. 3a, b)^26^. However, such enrichment was not observed in CHIP (+)-specific upregulated genes (Supplementary Fig. 3c), suggesting the presence of additional factors establishing CHIP (+)-specific immune responses in severe COVID-19.

To delineate CHIP (+)-specific immunological signatures, we conducted a direct comparison between CHIP (+) and CHIP (-) severe COVID-19 at individual cell-type resolution (Supplementary Table 6, see Methods). The enrichment analysis of DEGs based on cytokine-responsive gene sets revealed that classical monocytes exhibited CHIP (+)-specific strong immune responses compared to CHIP (-) (Supplementary Fig. 4a). In contrast, other cell types did not show a similar trend (Supplementary Fig. 4b-i). CHIP (+) upregulated genes in classical monocytes demonstrated more enriched inflammatory cytokine responses such as IL-1β (Mann‒Whitney’s *U* test, *P* = 3.24e-2, IL-1β) (Fig. 1e) and an elevation of other COVID-19 representative interleukins such as IL-6, IL-10, and IL-15 (Fig. 1f) compared to CHIP (-) upregulated genes^35^. Notably, CHIP (+) upregulated genes were uniquely associated with type II interferon (IFN) responses, which was not shown in CHIP (-) upregulated genes (Mann‒Whitney’s *U* test, *P* = 1.08e-3, IFN-γ) (Fig. 1e), potentially suggesting that a strong IFN-γ response in classical monocytes is a representative immunological signature in CHIP (+) severe COVID-19.

### Pseudotime analysis reveals IFN-γ-mediated hyperinflammation in CHIP (+) severe COVID-19

Since a high level of IFN-γ has been reported as an indicator of severe COVID-19 and is known to exacerbate inflammatory signatures^5,36,37^, we hypothesized that the strong IFN-γ response in CHIP (+) severe COVID-19 patients could be attributed to the hyperinflammation that occurs in severe COVID-19. To determine whether a strong IFN-γ response is associated with the progression of COVID-19 severity in CHIP (+), we conducted a pseudotime analysis in classical monocytes using specimens collected twice from one patient, excluding innate individual biases (Fig. 2a, see Methods). After ordering the cells along with the trajectory analysis, we allocated the annotation of high and low inflammation clusters based on inflammatory signatures termed in MsigDB Hallmark 2020 (Fig. 2b, Supplementary Table 7). We found that IFN-γ response genes were significantly enriched in the high inflammation group of CHIP (+), even more potent than those in the CHIP (-) group (Fig. 2c), again confirming that a strong IFN-γ response is a hallmark of CHIP (+) COVID-19 severity.

We further validated the immunological signature in CHIP (+) with previously reported IFN-γ-induced inflammation in COVID-19. Recent studies based on in vivo mouse experiments and scRNA-seq analyses of COVID-19-affected lungs have highlighted the role of the IFN-γ-induced inflammatory macrophage phenotype and the synergistic effect of IFN-γ and TNF-α in severe COVID-19^38,39^. Consistently, in our analysis, upregulated genes in the CHIP (+) high inflammatory cluster were significantly enriched by proinflammatory M1 macrophage-specific genes (Fig. 2d and Supplementary Fig. 5)^40,41^ and were associated with the synergistic inflammation signature through cotreatment with TNF-α and IFN-γ (Fig. 2e)^39^. Thus, the previously discovered IFN-γ-mediated disease-exacerbating mechanism explains how the IFN-γ response in classical monocytes uniquely contributes to hyperinflammation in CHIP (+) severe COVID-19 patients.

### Validation of the IFN-γ-mediated hyperinflammation signature in CHIP (+) severe COVID-19

**Fig. 3:**
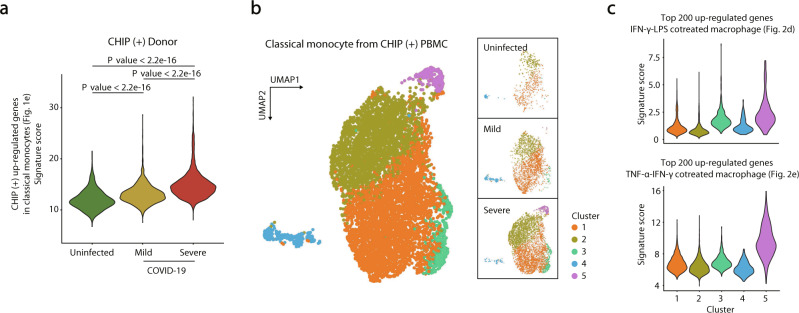
Validation of the IFN-γ signature according to COVID-19 severity in CHIP (+) individuals. **a** Violin plots presenting distributions of signature scores in individual cells for CHIP (+) upregulated genes in classical monocytes (Fig. 1e). CHIP (+) uninfected donors, *n* = 482; CHIP (+) mild COVID-19, *n* = 2163; CHIP (+) severe COVID-19, *n* = 4408. A two-sided Kolmogorov‒Smirnov test was performed. **b** UMAP showing subclusters in classical monocytes from all CHIP (+) individuals (left), uninfected donors (top right), mild COVID-19 (middle right), and severe COVID-19 (bottom right). **c** Violin plots showing the distributions of scores for DEGs between IFN-γ-LPS and untreated human macrophages (top) and DEGs between TNF-α-IFN-γ cotreated and untreated conditions (bottom) in each cell from each cluster. Cluster 1, *n* = 3543; Cluster 2, *n* = 2636; Cluster 3, *n* = 385; Cluster 4, *n* = 272; Cluster 5, *n* = 217.

To determine whether a potent IFN-γ response is an indicator of COVID-19 severity in patients with CHIP, we examined the expression patterns of CHIP (+) upregulated genes in classical monocytes (Fig. 1e) across CHIP (+) uninfected donors, mild, and severe COVID-19 patients. We found that these genes were gradually upregulated according to COVID-19 severity in CHIP (+) individuals (Fig. 3a). Notably, such a trend was not observed in CHIP (-) individuals, confirming that the potent IFN-γ response is a unique immune signature of COVID-19 severity in CHIP (+) individuals (Supplementary Fig. 6a).

Next, we sought to identify unique subcellular populations according to COVID-19 severity in CHIP (+) individuals. We performed a subclustering analysis using classical monocytes from all CHIP (+) individuals and revealed a unique cell population in severe COVID-19 patients with CHIP (Fig. 3b). Of the 5 clusters, Cluster 3 and Cluster 5 were enriched in CHIP (+) severe COVID-19 patients (Supplementary Fig. 6b). Interestingly, the genes associated with proinflammatory M1 macrophages were highly expressed in both Cluster 3 and Cluster 5, representing systemic inflammation in severe COVID-19 patients (Fig. 3c). Additionally, Cluster 5 showed a synergistic inflammatory signature through cotreatment with TNF-α and IFN-γ, implying an association with COVID-19 severity. Collectively, the combined immune response of IFN-γ and TNF-α was a hallmark of COVID-19 severity in CHIP (+) individuals.

### *DNMT3A* CHIP mutation-dependent hypo-DMRs are linked to CHIP (+) specific response genes

**Fig. 4:**
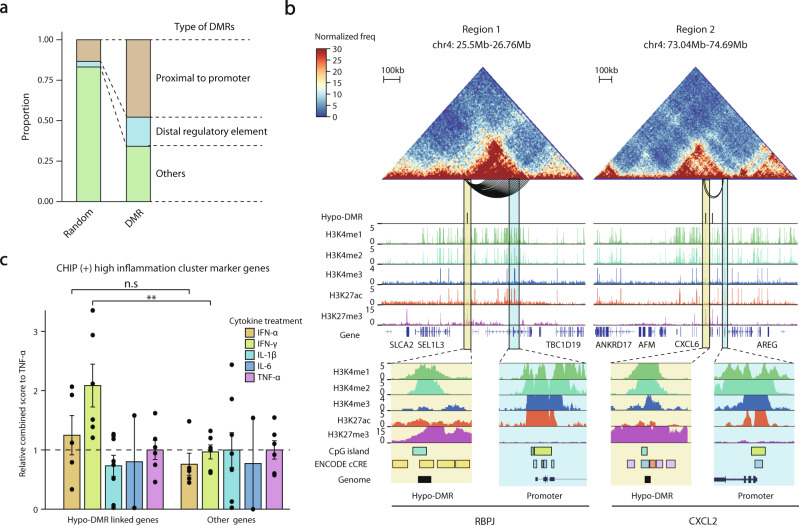
Regulatory potential of *DNMT3A* CHIP-specific hypo-DMRs in IFN-γ response genes. **a** Stacked bar plots showing the proportion of annotated hypo- and hyper-DMRs. In DMRs: proximal to the promoter, *n* = 1124; distal regulatory element, *n* = 423; others, *n* = 801. In randomly selected DMRs: proximal to the promoter, *n* = 314; distal regulatory element, *n* = 81; others, *n* = 1953. **b** Hi-C heatmap and other regulatory features for two example regions. Top, Hi-C contact maps (heatmap) for region 1 (*RBPJ*) and region 2 (*CXCL2*) with significant long-range chromatin interactions with the promoter regions and hypo-DMRs (arcs below the heatmap). Middle, distribution of hypo-DMRs and histone modification signals along Hi-C contact maps. Bottom, snapshots of interacting regions in the UCSC genome browser^58^. Each DMR and promoter region are highlighted in yellow and blue, respectively. On the CpG island track, the color indicates the length of the islands: light green, smaller than 300 bases; cyan, larger than 300 bases. On the ENCODE cCRE track, the color indicates the type of signature of the DNA element: yellow, distal enhancer-like signature; orange, proximal enhancer-like signature; blue, promoter-like signature; purple, Dnase-H3K4me3 signature. For promoter regions, neither distal nor proximal enhancer signatures are shown in the figure. **c** Bar plots of combined scores relative to TNF-α for hypo-DMR linked genes and others for the same gene ontology library in Fig. 2c. Each point indicates a single-ligand perturbation term. The mean and standard error of the mean (SEM) of each gene set are presented in a bar plot. A one-sided Mann‒Whitney’s *U* test was performed (n.s.: nonsignificant, ^**^P < 0.01).

We sought to identify the underlying gene regulation mechanisms of IFN-γ-mediated exacerbation of inflammation in CHIP (+) severe COVID-19. Given that CHIP is driven by mutations in multiple epigenetic regulators, such as *DNMT3A*, *TET2*, and *ASXL1*, we hypothesized that altered chromatin activity might play a critical role in facilitating IFN-γ response gene expression in severe COVID-19. To test our hypothesis, we first identified CHIP-specific 2348 differentially methylated regions (DMRs) by using acute myeloid leukemia patients carrying *DNMT3A* CHIP mutations (Supplementary Table 8, see Methods)^42^. In support of our hypothesis, these DMRs highly overlapped with putative *cis*-regulatory elements (Fisher’s exact test, *P* < 0.001 for proximal to the promoters; *P* < 0.001 for distal regulatory elements) (Fig. 4a, see Methods).

As many *cis*-regulatory elements are known to target genes over large genomic distances^43^, we precisely annotated target genes of the DMRs by performing in situ Hi-C experiments on CD14^++^/CD16^−^ classical monocytes of two healthy donors (see Methods)^44^. Using this information, as illustrated for the *RBPJ* and *CXCL2* genes (Fig. 4b and Supplementary Fig. 7a), we revealed that, in total, approximately 33% of CHIP (+) upregulated genes in classical monocytes of severe COVID-19 patients were associated with hypo-DMRs either in proximal (within 15 kb) or long-range chromatin interactions (over 15 kb but less than 2 Mb) denoted as ‘hypo-DMR linked genes’ (Supplementary Fig. 7b). Notably, hypo-DMR linked genes largely overlapped with IFN-γ response genes (Mann‒Whitney’s *U* test, *P* = 1.08e-3, IFN-γ), which was not shown in the other remaining genes (Fig. 4c). Further histone H3 27th lysine acetylation (H3K27ac) peaks of IFN-γ-LPS-stimulated human classical monocytes^32^ were enriched in the linked hypo-DMRs (Fisher’s exact test, *P* = 4.92e-7 for replicate 1; *P* = 1.43e-11 for replicate 2) (Supplementary Fig. 8a, b) but not in other immune cell types (Supplementary Fig. 8c, d). Taken together, our results indicate that CHIP-dependent altered chromatin activities of *cis*-regulatory elements may facilitate IFN-γ-mediated hyperinflammation response gene expression of classical monocytes in CHIP (+) severe COVID-19 patients.

### Activation of poised *cis*-regulatory elements primes CHIP (+) specific IFN-γ response genes

**Fig. 5:**
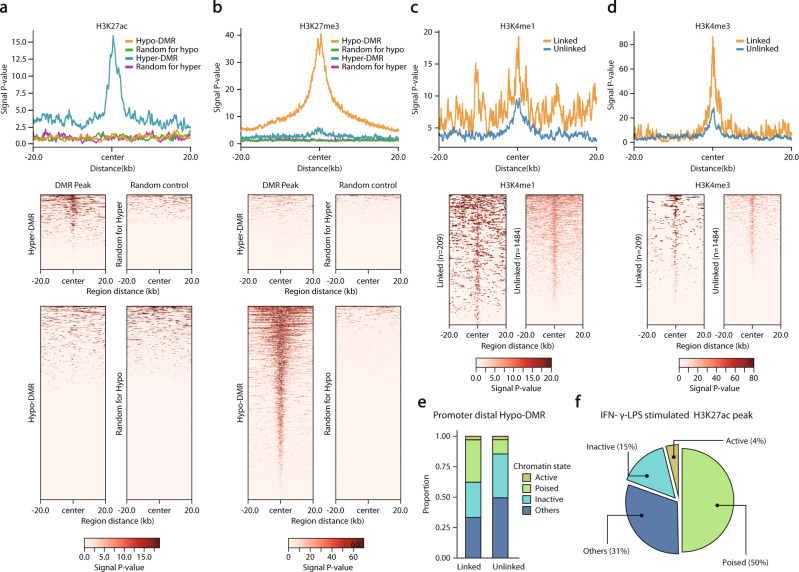
Chromatin states of hypo-DMRs linked to CHIP specifically upregulated genes. **a**, **b** ChIP-seq signal distribution (**a**) for H3K27ac and (**b**) for H3K27me3 of 20k upstream and downstream surrounding hypo-DMRs (*n* = 1693), hyper-DMRs (*n* = 655), and randomly selected regions. Each randomly selected region has the same distribution of chromosome number and length of hypo- and hyper-DMRs, respectively. Top, average ChIP-seq signal distributions. Bottom, heatmaps of ChIP-seq signals of the corresponding regions. **c**, **d** ChIP-seq signal distribution for CHIP (+) upregulated genes linked (*n* = 209) and unlinked (*n* = 1484) hypo-DMRs for H3K4me1 and H3K4me3. Top, average profiles of ChIP-seq signals for linked and unlinked hypo-DMRs. Bottom, heatmaps of ChIP-seq signals of the corresponding regions. **e** Stacked bar plots of the linked and unlinked hypo-DMRs with the annotation of chromatin states. The plots show annotations of hypo-DMRs distal to promoters. **f** A pie chart showing the proportions of chromatin states of promoter distal hypo-DMRs intersected with H3K27ac ChIP-seq peaks in IFN-γ/LPS-stimulated human macrophages. For statistical significance, a one-sided Fisher’s exact test was performed to compare each annotated hypo-DMRs and the remaining promoter-distal hypo-DMRs for the enrichment of H3K27ac peaks.

**Fig. 6:**
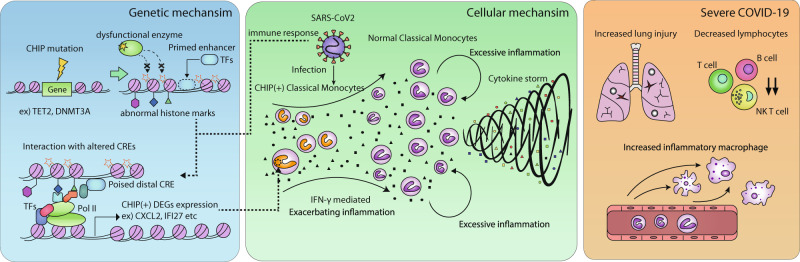
A proposed model for the pathogenesis of severe COVID-19 in patients with CHIP. A schematic overview showing the progression of disease exacerbation in terms of gene regulation, cellular levels, and clinical/immunological signatures in CHIP (+) severe COVID-19.

To elucidate how ‘linked hypo-DMRs’ facilitate IFN-γ response gene expression in COVID-19 patients with CHIP, we examined the regulatory potential of hypo-DMRs based on four representative histone modification marks of primary human classical monocytes: histone H3 4th lysine monomethylation (H3K4me1) and trimethylation (H3K4me3) and 27th lysine acetylation (H3K27ac) and trimethylation (H3K27me3)^45,46^. When comparing DMRs to randomly selected genomic regions, hypo-DMRs were mostly marked by H3K27me3 in primary human classical monocytes, while hyper-DMRs were enriched in H3K27ac as an indicator of active *cis*-regulatory elements (Fig. 5a, b). However, interestingly, a subset of hypo-DMRs linked to CHIP (+) severe COVID-19 upregulated genes (Supplementary Fig. 7b) was also significantly co-occupied by H3K4me1 and H3K4me3 peaks compared to the unlinked hypo-DMRs (Fisher’s exact test, *P* < 0.001, H3K4me1; *P* < 0.001, H3K4me3) (Fig. 5c, d). Such a coexhibition of inactive and active chromatin signatures may indicate that the regulatory elements of CHIP (+) upregulated genes shifted from poised or bivalent status to an active chromatin state through the process of CHIP-dependent DNA hypomethylation. To test this possibility, we annotated the chromatin states of linked hypo-DMRs according to the combination of histone modification marks (Fig. 5e and Supplementary Fig. 8e, see Methods). We found that only poised status (H3K4me1 peak without both H3K27me3 and H3K27ac peak, putative poised enhancers) was significantly enriched by linked hypo-DMRs compared to unlinked hypo-DMRs (Fisher’s exact test, *P* = 4.76e-06), supporting that the altered chromatin activity of poised *cis*-regulatory elements is associated with IFN-γ response gene expression (Fig. 5e). Furthermore, H3K27ac peaks induced by IFN-γ-LPS stimulation were also enriched in the poised status compared to the remaining chromatin states (Fisher’s exact test, *P* = 3.63e-2), again confirming the role of poised *cis*-regulatory elements in the activation of IFN-γ response genes (Fig. 5f). Thus, CHIP mutants appear to reprogram epigenetic states, including DNA hypomethylation at poised enhancers, which primes IFN-γ-associated immune response genes, thereby driving hyperinflammation and leading to a critical course of COVID-19 (Fig. 6).

## Discussion

By profiling the gene expression of severe COVID-19 in the presence or absence of CHIP through a single-cell technique, we revealed a distinct IFN-γ-mediated immune signature in CHIP (+) severe COVID-19, which was partly explained by CHIP-dependent chromatin reorganization. Our results strongly indicate that CHIP may play a critical role in the progression of severe COVID-19 through its own immunological pathways.

Individuals with COVID-19 have been reported to have heterogeneous presentations ranging from asymptomatic to critical illness^2^. In line with this, many studies divided COVID-19 patients into subgroups defined by immunological characteristics, such as patterns of sepsis^47^, subpopulations of lymphocytes^48^, IFN responses in the lung^49^, or loss-of-function variants^50^. Single-cell techniques have been vigorously applied in COVID-19 to dissect the underlying causes of the diverse immune responses^37,51,52^ and to elaborate on the relationship between immune subtypes and clinical characteristics^53,54^. Despite these important studies, none of the single-cell studies has characterized the immunological effects of CHIP in COVID-19. In this regard, the current study uniquely demonstrated how CHIP-associated somatic mutations in immune cells could be used to establish a novel subgroup of COVID-19 patients.

Single-cell immune transcriptome analysis revealed that IFN-γ-related hyperinflammation is a hallmark of CHIP (+) severe COVID-19. In particular, there was an enrichment of the inflammatory signature in classical monocytes, which is compatible with recent knowledge regarding the effect of CHIP on myeloid-skewed hematopoietic stem cell differentiation^55^. From a cytokine perspective, IFN-γ and its synergism with TNF-α were thought to play a critical role in the pathogenesis of severe COVID-19 in CHIP (+) patients. This finding aligns with a previous report stating the role of IFN-γ and/or TNF-α in exacerbating chronic inflammatory disease by CHIP^55^. Our study also implies that both type I IFN and type II IFN responses play an important role in disease exacerbation in certain patients with severe COVID-19. An additional interesting immunological finding reasonably explained by CHIP biology is the upregulation of genes related to inflammatory macrophages in CHIP (+) severe COVID-19. CHIP is well known to drive hyperinflammation in chronic diseases, which is mainly attributable to the altered function of monocytes and macrophages^55^.

In this study, we provided a potential gene regulation mechanism under CHIP-dependent altered chromatin activities, but the characterization of chromatin status was limited to AML patients carrying *DNMT3A* CHIP mutations. Nevertheless, we found that hypo-DMRs were enriched by active or poised *c*REs in IFN-γ-LPS-stimulated or primary human classical monocytes, respectively, supporting the notion that CHIP-specific chromatin alterations may involve IFN-γ-induced hyperinflammation in CHIP (+) severe COVID-19. Additional examinations between CHIP-driven chromatin reorganization and disease susceptibility under various infections are needed in future studies.

In terms of therapeutic strategies, due to the significant implication of type I and type II IFN responses in severe COVID-19, anti-inflammatory strategies targeting not only inflammatory cytokines but also pathological IFN responses need to be investigated. Notably, in CHIP (+) severe COVID-19, we noticed that a spleen tyrosine kinase (Syk) inhibitor, which is known to reduce the expression of interferon-stimulated genes^56^, might be a molecule that is effective in suppressing the pathogenic immune responses induced by CHIP. An in vitro study on the Syk inhibitor fostamatinib suggested its therapeutic effect against COVID-19^57^, and we have shown the results of a randomized placebo-controlled trial with the drug (NCT04579393).

In conclusion, we successfully elucidated a unique CHIP-driven immunological mechanism in severe COVID-19. Revealing the underlying epigenetic mechanism for the altered immune function that aligns with well-known CHIP biology suggests the robustness of our findings. It appears that classical monocytes in patients with CHIP (+) COVID-19 undergo distinct immune responses; thus, studies focusing on immunomodulation strategies based on the presence of CHIP are needed. Considering the shared pathogenic host immune response across infections, we postulate that our findings might provide a better understanding of the previously unexplained exacerbation of clinical conditions by various viruses in patients with CHIP.

## Supplementary information

Supplementary Figures, Methods and Legends for supplementary data

Supplementary information 1. Supplementary Tables

## Data Availability

Readers are welcome to comment on the online version of the paper. Correspondence and requests for materials should be addressed to I.J. (ijung@kaist.ac.kr) or Y.Koh. (snuhgo01@gmail.com). Source data are provided with this paper. All scRNA-seq data generated and/or analyzed during the current study are available in the Gene Expression Omnibus data repository with accession number GSE182123 and will be available after publication. All published raw data used in the analysis are available through accession numbers or identifiers written in the Methods and/or Supplementary information. We used publicly available software for all analyses and followed standardized processes. No custom code was used for the analyses performed in this study. Supplementary information is available at *Experimental & Molecular Medicine*’s website.
