# Supplementary material for: Single-cell transcriptome analyses reveal distinct gene expression signatures of severe COVID-19 in the presence of clonal hematopoiesis
Source: Exp Mol Med. 2022 Oct 13;54(10):1756–65. doi: 10.1038/s12276-022-00866-1 (PMC9559247; doi:10.1038/s12276-022-00866-1)

Supplementary Methods

Principal component analysis of variable gene expression among disease groups at cell-type resolution

In Fig. 1c, the raw UMI counts were merged separately for each cell type according to the healthy donor, influenza, CHIP (-) mild COVID-19, CHIP (-) severe COVID-19, CHIP (+) mild COVID-19, and CHIP (+) severe COVID-19. Dividing by the total UMI counts in each cell type and multiplying by 100 000, we normalized the merged UMI counts for each gene as the normalized gene expression. To analyze the dysregulation of gene expression in diseases compared to the healthy donors, the normalized gene expression was divided by the values in the healthy donor group. Moreover, highly variable genes across cell types and diseases were identified based on the top 25% standard deviation. We log-transformed the data (pseudo-count=1) and calculated the spearman's correlation coefficients. PCA was performed using the correlation coefficients and the projections of PC1 and PC2 were visualized in Fig. 1c.

Trajectory analysis

In Fig. 2a-c, Trajectory analysis was applied to double sampled two severe COVID-19 patients using Monocle2 (v2.18.0)[1]. By applying FindVariableFeatures function with vst method in Seurat R package, 2 000 highly variable genes were calculated in classical monocytes across each patient (CHIP (+) patient, CHIP_sev_1_1 and CHIP_sev_1_2; CHIP (-) patient- nCoV 7 and nCoV 8, names of samples listed in Supplementary Table 1). newCellDataSet function was used to create UMI count matrix of the highly variable genes with default parameters, except expressionFamily = "negbinomial.size". After estimation of size factors and dispersion of gene expression with estimateSizeFactors and

estimateDispersions, dimension reduction was done on normalized count matrix by DDRTree algorithm based reduceDimension function with default parameters, except scaling = FALSE. After clustering and aligning cells along pseudotime trajecotry with orderCells, marker genes for each cluster were identified using MAST algorithm. The smallest cluster was excluded in the following analysis for both CHIP (+) and CHIP (-). Based on size factor and dispersion normalized expression level, the expression pattern of marker genes was plotted along aligned cells using plot_pseudotime_heatmap fuction.

Single-cell RNA-seq analysis for M1 polarized macrophage

Supplementary Fig. 5 used DEGs between M1 polarized macrophage and M0 naïve macrophage (GSE164498)[2]. It provides scRNA-seq data of HL-60 derived macrophage differentiating to M1, with IFN-γ and LPS treatment from 0 hours to 24 hours. The raw count data of 0hrs treatment (GSM5012287), 3hrs treatment (GSM5012288), 6hrs treatment (GSM5012289), 12hrs treatment (GSM5012290), 24hrs treatment (GSM5012291) were converted into Seurat object with Seurat R package (v4.0.1)[3], excluding cells or genes that expressed <200 genes or were not expressed in any cells. 0hrs treatment sample was annotated as M0 macrophage and rest of 4 treatment samples were annotated as M1 macrophage. The number of M0 cells was 4 038 and the number of M1 cells was 1 766. Between M0 and M1, DEG identification was made following the same process in ‘Identification of DEGs using MAST’.

Single-cell RNA-seq analysis for TNF-α-IFN-γ stimulation and untreated macrophages

Fig. 2e used DEGs between TNF-α-IFN-γ stimulation and untreated macrophages (GSE168710)[4]. Four human donors’ blood-derived macrophages were stimulated by 9 distinct types of cytokine conditions including untreated condition and TNF-α- IFN-γ

cotreatment, and undergone single-cell RNA sequencing. Filtered feature barcode matrices of four samples (GSM5159500, GSM5159501, GSM5159502, GSM5159503) were downloaded in h5 format from GEO database and integrated into Seurat object, including cells or genes that expressed at least 1 gene in 1 cell. The identity of the stimulation condition was determined according to metadata contained in downloaded data. DEG identification was done between TNF-α-IFN-γ cotreatment and untreated, following the same process in 'Identification of DEGs using MAST'.

Gene set enrichment analysis

Gene set enrichment analysis (GSEA) was applied to DEGs with GSEA program (v4.1.0) (Fig. 2d, e and Supplementary Fig. 3a-c, 5)[5,6]. GSEA was run with 'Run GSEAPreranked' tools in the program. Pre-ranked gene list was ranked with a decreasing order based on log2 fold change between two different conditions. Gene sets for enrichment test were prepared in gmx format, and compared to pre-ranked list with a number of permutations 1 000, no collapse to gene symbol. After analysis, GSEA provided normalized enrichment score (NES), nominal p-value, FDR q-value, FWER p-value as normalization statistics. In all figures, NES and FDR q-values are presented. For Fig. 2d and 2e, DEGs between inflammation clusters were used as a pre-ranked list for both CHIP (+) and CHIP (-) severe COVID-19 patients in classical monocytes. Log2 fold changes of DEGs were calculated by FindMarkers based on low inflammation cluster, positive value for high inflammation cluster up-regulated genes and negative value for low inflammation cluster ones. Each IFN-γ-LPS vs untreated and TNF-α-IFN-γ vs untreated DEGs in human macrophage was prepared in gmx format for Fig. 2d and 2e, respectively. For Supplementary Fig. 3a-c, DEGs of CHIP (+) and CHIP (-) compare to CHIP (-) Mild COVID-19 group for each immune cell types were aggregated and used as a pre-ranked list. Log2 fold changes of DEGs were calculated by FindMarkers based

on CHIP (-) Mild COVID-19 group for each cell types and disease group. Average of calculated log2 fold changes except missing values were used for making pre-ranked list. In Supplementary Fig. 3a, commonly up- and down-regulated genes in severe group were pre-ranked according to averaged fold changes of including disease states, CHIP (+) and CHIP (-). In Supplementary Fig. 3b, CHIP (-) specific and Mild up-regulated genes were selected from DEGs between CHIP (-) severe and Mild group and pre-ranked according to averaged fold changes, with negative value for Mild up-regulated genes. In a same manner, CHIP (+) specific and Mild up-regulated genes were pre-ranked with averaged fold changes in Supplementary Fig. 3c. A gene set associated with Flu and Severe COVID-19 in previous study [7] was prepared in gmx format for all GSEA analyses in Supplementary Fig. 3. In Supplementary Fig. 5, it follows same process as Fig. 2d, 2e, except using M1 vs M0 macrophage DEGs[2] in gmx format.

Identification of differentially methylated regions associated with *DNMT3A* mutations

To define DMRs associated with *DNMT3A* mutations, we first obtained whole-exome sequencing data for 94 public AML samples downloaded from dbGaP under accession phs001027.v2.p1. Exome sequencing reads were aligned to hg38 reference genome with bwa v0.7.17, and somatic variants called by at least one of the two callers, Strelka2 v2.9.7 and Varscan v2.4.3, were considered as valid somatic variants. The resulting variants were annotated with SnpEff v4.3.1. To reduce false-positive calls, we only considered variants meeting the following criteria: (1) variant allele frequency greater than 5%, (2) at least five sequencing reads supporting the variant, [3] ExAC allele frequency less than 1%, and (4) only missense, nonsense, frameshift and splice variants. For public AML data, 33 out of 94 samples had *DNMT3A* mutations. Of the 39 identified *DNMT3A* mutations, 27 were previously reported to be associated with clonal hematopoiesis [8].

Next, we downloaded 94 public enhanced reduced-representation bisulfite sequencing data of the matched AML patients from dbGaP under accession phs001027.v2.p1. Trim galore! v0.6.4_dev with –rrbs option was used for the adapter-trimming of raw sequencing reads, and the resulting reads were aligned to the hg38 reference genome with Bismark v0.22.3. Methylation levels at CpG context were computed using MethylDackel. To control the levels of noise in methylation levels, we restricted the downstream analyses to the set of CpGs covered by at least 10 aligned reads. Using processed methylation levels, DMRs were called between 33 AML samples with *DNMT3A* mutations and 61 AML samples without *DNMT3A* mutations by Metilene v0.2-8. Maximum distance allowed between two CpGs in a DMR was set to 500bp, and at least four CpGs were required within a region to be called as a DMR. DMRs with Benjamini-Hochberg corrected p-values less than 0.05 were finally called as DMR.

Processing of Hi-C data

Raw fastq files were mapped to hg38 reference genome by BWA-mem function using '–M' option. Among aligned reads, the following reads were filtered out-Low quality reads (MAPQ < 10), reads spanning ligation sites, chimeric reads, trans reads and self-interacting reads (two fragments located within 15kb). Pairs of filtered reads were merged to have a form of paired-end aligned BAM files. PCR duplicates were removed with Picard. To remove experimental and intrinsic biases covNormRpkg R package (v1.1.0) was used[9]. In brief, coverage and frequency of the BAM file on 10kb resolution were calculated with coverageBed in bedtools (v2.29.0) with -counts, -sorted option and used as an input. Then a list of coverage and frequencies were filtered by filterInputDF function. Filtered data were then undergone coverage normalization by normCoverage function with a default option. To

calculate significant interactions in Hi-C data, distance normalization steps were implemented on coverage normalized data using normDistance, set maximal distance option as 2 000 000. After distance normalization, interaction that has p-value < 0.05 and FDR< 0.1 were regarded as significant interactions.

Analysis of histone ChIP-seq results in classical monocytes

To annotate regulatory roles of hypo-DMRs, ChIP-seq data for each histone modifications were collected from the ENCODE portal (https://www.encodeproject.org/)[10]. Three distinct types of data were downloaded and used for Fig. 4, 5 and Supplementary Fig. 8. Fold change over control bigwig files were downloaded for visualization purposes. Signal p-value bigwig files were downloaded and used for making averaged profile and peak heatmap through Deeptools (v3.5.1) in Fig. 5a-d. Each profile and heatmap was respectively drawn by plotProfile and plotHeatmap functions, based on matrix of scores per genome region calculated by computeMatrix function, --referencepoint=center, with 20kb as upstream and downstream option. Narrow peak bed files were downloaded for annotation of chromatin states in Fig. 5e and Supplementary Fig. 8e. Identifiers of the downloaded data are the following. For fold change over control, H3K4me1, ENCFF135CCA; H3K4me2, ENCFF040JVY; H3K4me3, ENCFF739LQS; H3K27ac, ENCFF116NCG; H3K27me3, ENCFF759NGN. For signal p-value, H4K3me3: ENCFF296KZJ, ENCFF810NAR, ENCFF704SGA; H3K4me1: ENCFF446EKY, ENCFF869JYG, ENCFF944QWS; H3K27ac: ENCFF626UTP, ENCFF552MVN, ENCFF245JNF; H3K27me3: ENCFF199TKI, ENCFF702ZRF, ENCFF335LFA. For narrow peak bed file, H4K3me3: ENCFF834FGQ, ENCFF061HWH, ENCFF102ZIF; H3K4me1: ENCFF928CYN, ENCFF240OSC,

ENCFF751KSU; H3K27ac: ENCFF505MRR, ENCFF471QGU, ENCFF506FIB; H3K27me3: ENCFF082UWB, ENCFF623KKM, ENCFF414EHN.

To observe the enrichment patterns of active signals in DMRs when inflammation occurs, two different replicates of ChIP-seq data for H3K27ac peaks of IFN-γ-LPS treated human macrophage were used (GSM1057019, replicate 1; GSM1057023, replicate 2)[11]. Processed wig data were downloaded from GEO database and genomic coordinates of these data were converted from hg19 to hg38 using CrossMap (v0.5.2)[12] with hg19tohg38 chain file provided in UCSC genome browser, producing outputs in a bigwig format. These bigwig files were used for making profile and peak heatmap through Deeptools (v3.5.1) in Supplementary Fig. 8a and 8b with –missingDataAsZero option. Similarly, processed peak data were also downloaded from GEO database and the genomic coordinates of the data were converted from hg19 to hg38 using CrossMap (v0.5.2), producing outputs in a bed format. With intersectbed in bedtools (v2.29.0), hypo-DMRs intersected with the peak bed files were listed. The number of intersection between hypo-DMRs and H3K27ac peaks was used for performing fisher’s exact test.

References

1. Qiu, X. *et al.* Single-cell mRNA quantification and differential analysis with Census. *Nat. Methods* **14**, 309-315 (2017).
2. Carvalho K., R. E., Jansen C., Williams K., Dowey A., McGill C., Mortazavi A. Uncovering the Gene Regulatory Networks Underlying Macrophage Polarization Through Comparative Analysis of Bulk and Single-Cell Data. Preprint at https://doi.org/10.1101/2021.01.20.427499 (2021).
3. Hao, Y. *et al.* Integrated analysis of multimodal single-cell data. *Cell* **184**, 3573-3587 e3529 (2021).
4. Zhang, F. *et al.* IFN-gamma and TNF-alpha drive a CXCL10+ CCL2+ macrophage phenotype expanded in severe COVID-19 lungs and inflammatory diseases with tissue inflammation. *Genome Med.* **13**, 64 (2021).
5. Mootha, V. K. *et al.* PGC-1alpha-responsive genes involved in oxidative phosphorylation are coordinately downregulated in human diabetes. *Nat. Genet.* **34**, 267-273 (2003).
6. Subramanian, A. *et al.* Gene set enrichment analysis: a knowledge-based approach for interpreting genome-wide expression profiles. *Proc. Natl. Acad. Sci. U. S. A.* **102**, 15545-15550 (2005).
7. Lee, J. S. *et al.* Immunophenotyping of COVID-19 and influenza highlights the role of type I interferons in development of severe COVID-19. *Sci. Immunol.* **5**, eabd1554 (2020).
8. Watson, C. J. *et al.* The evolutionary dynamics and fitness landscape of clonal hematopoiesis. *Science* **367**, 1449-1454 (2020).
9. Kim, K. & Jung, I. covNorm: An R package for coverage based normalization of Hi-C and capture Hi-C data. *Comput. Struct. Biotechnol. J.* **19**, 3149-3159 (2021).
10. Davis, C. A. *et al.* The Encyclopedia of DNA elements (ENCODE): data portal update. *Nucleic Acids Res.* **46**, D794-D801 (2018).
11. Qiao, Y. *et al.* Synergistic activation of inflammatory cytokine genes by interferon-gamma-induced chromatin remodeling and toll-like receptor signaling. *Immunity* **39**, 454-469 (2013).
12. Zhao, H. *et al.* CrossMap: a versatile tool for coordinate conversion between genome assemblies. *Bioinformatics* **30**, 1006-1007 (2014).

Supplementary Tables

Supplementary Table 1. Clinical information of patient used in scRNA-seq

Table showing clinical information of patient used in analysis of scRNA-seq. Sequencing info indicates sample name of each sequencing data. CRP indicates C-reactive protein. Ordinal score is the severity of COVID-19, ranging from 1-8. Cases with the highest ordinal scale 3 or more were classified as severe COVID-19.

Supplementary Table 2. The list of CHIP mutations in CHIP (+) individuals

Table showing the information about CHIP mutations in CHIP (+) individuals. The information about the mutations such as associated genes, type of mutations, and variant allele frequency were provided.

Supplementary Table 3. Data quality of scRNA-seq

Table showing data quality of scRNA-seq. It contains existence of CHIP mutation and quantity of detected unique molecular identifiers and genes.

Supplementary Table 4. Information of individual cell in scRNA seq

Table showing the information of each cell such as origin of sample, disease of sample, annotated cell type, the number of detected molecules, and the number of detected genes.

Supplementary Table 5. Lists of genes from comparison between CHIP (+) and CHIP (-) severe COVID-19 upregulated-genes

Table showing the lists of genes from comparison between CHIP (+) and CHIP (-) severe COVID-19 upregulated-genes used in analysis for Fig. 1d.

Supplementary Table 6. DEG lists between CHIP (+) and CHIP (-) severe COVID-19 in each cell type

Table showing the DEG lists between CHIP (+) and CHIP (-) severe COVID-19 in each cell type. All lists were used in the analysis for Supplementary Fig. 4, while the DEG lists from classical monocytes were used in Fig. 1e.

Supplementary Table 7. DEG lists between subclusters from trajectory analysis in classical monocytes of CHIP (+) and CHIP (-) severe COVID-19

Table showing the DEG lists between subclusters from trajectory analysis in classical monocytes of CHIP (+) and CHIP (-) severe COVID-19 used in the analysis for Fig. 2b and c.

Supplementary Table 8. A list of DMRs

Table showing a list of whole differentially methylated regions (DMRs) between AML patients who had *DNMT3A* mutation and did not. Hypo-DMR and hyper-DMR were classified according to mean_difference column. DMR was classified to hypo-DMR if mean_difference is positive, and classified to hyper-DMR if negative.

Supplementary Figure Legends

Supplementary Fig. 1: Quality-control of single-cell RNA-seq results

a, **b** Scatter plots showing CHIP (+) individuals' immune cells according to UMI count and other features. **a** Percentage of mitochondrial genes **b** the number of detected genes. **c** Scatter plot of log 10-transformed count data for CHIP (+) individuals. R represents Pearson's correlation coefficient values. **d** Pattern of cell distributions from each individual in UMAP plot.

Supplementary Fig. 2: Cell-type annotation of single-cell RNA-seq results

a UMAP clusters of integrated single-cell data. **b** Expression patterns of representative marker genes of immune cells represented by dot plot. **c** Stacked bar plots showing the cell-type proportions of each individual.

Supplementary Fig. 3: GSEA analyses of DEGs of CHIP (+) and CHIP (-) severe COVID-19 compared to mild COVID-19

a-c GSEA plots with a gene set associated with Flu and Severe COVID-19, showing high TNF-α/IL-1β signature. Genes are ordered based on averaged log-fold changes. Normalized enrichment scores (NES) and FDR are presented for the subsets of DEGs between CHIP (+) and CHIP (-) severe COVID-19 compared to CHIP (-) mild COVID-19 group, respectively. **a** Common up- and down-regulated genes. **b** CHIP (-) specific and mild COVID-19 up-regulated genes. **c** CHIP (+) specific and mild COVID-19 up-regulated genes.

Supplementary Fig. 4: Distinct cytokine responsive signatures between CHIP (+) and CHIP (-) severe COVID-19

a-i Scatter plots showing combined scores of differentially expressed genes (DEGs) in selected cell types between severe COVID-19 patient groups for the same gene ontology library in Fig. 2c. The horizontal axis, up-regulated genes in CHIP (+) patients; vertical axis, up-regulated genes in CHIP (-) patients. The identity lines are presented diagonally. The color indicates the type of perturbed ligand in database terms. **a** Classical monocytes. **b** non-EM-

like CD8+ T cells. **c** EM-like CD8+ T cells. **d** non-EM-like CD4+ T cells. **e** NK cells. **f** nonclassical monocytes. **g** IgG+ B cells. **h** IgG- B cells. **i** EM-like CD4+ T cells

Supplementary Fig. 5: The immunological characteristics of CHIP (+) high inflammation cluster up-regulated genes

GSEA plots for marker genes of inflammation clusters in CHIP (+) and CHIP (-), respectively. Genes are ordered based on log-fold changes between high inflammation cluster and low inflammation cluster. Normalized enrichment scores (NES) and FDR are presented for DEGs between M1 and M0 macrophage.

Supplementary Fig. 6: Distinct signatures of IFN-γ response according to COVID-19 severity

a The distributions of scores in each cell for CHIP (+) up-regulated genes classical monocytes (Fig. 1e) represented by violin plots according to the COVID-19 severity in CHIP (-). Healthy donors, n=3 065; CHIP (-) mild COVID-19, n=2 777; CHIP (-) severe COVID-19, n=1 585. Two-sided Kolmogorov-Smirnov test was performed. **b** The cell proportion of each subcluster from classical monocytes within each severity group of CHIP (+) donors.

Supplementary Fig. 7: Analyzes of long range interaction between hypo-DMRs and DEGs of CHIP (+) severe COVID-19 in classical monocyte

a Examples of analysis of Hi-C data. A whole Hi-C interaction map of chromosome 4 at 40kb resolution. The color indicates normalized chromatin contact frequencies. Two example

regions containing CHIP (+) severe COVID-19 up-regulated genes in classical monocytes (regions 1 and 2) are highlighted by yellow dashed lines. **b** A pie chart showing the proportions of hypo-DMR linked up-regulated genes in CHIP (+) severe COVID-19 classical monocytes.

Supplementary Fig. 8: Analyses of DMRs coupled with immune signatures and histone modifications

a, **b** ChIP-seq signal distribution of 20k upstream and downstream surrounding CHIP (+) up-regulated genes linked (n=209) and unlinked (n=1 484) hypo-DMRs for H3K27ac signal of two different replicates. Top, average profiles of ChIP-seq signals for linked and unlinked hypo-DMRs. Bottom, heatmaps of ChIP-seq signals of the corresponding regions. **a** Replicate 1 (GSM1057019). **b** Replicate 2 (GSM1057023). **c**, **d** Bar plots of combined scores of CHIP (+) up-regulated genes linked to hypo-DMRs in 9 immune cell types for the same gene ontology library in Fig. 4c. Each point indicates a single-ligand perturbation term. The mean and standard error of the mean (SEM) of each gene set are presented in a bar plot. **c** IFN-α perturbation, **d** IFN-γ perturbation. **e** Stacked bar plots of the linked and unlinked hypo-DMRs with annotation of chromatin states. It shows annotations of hypo-DMRs proximal to promoters.

Choi et al Supplementary Fig. 1

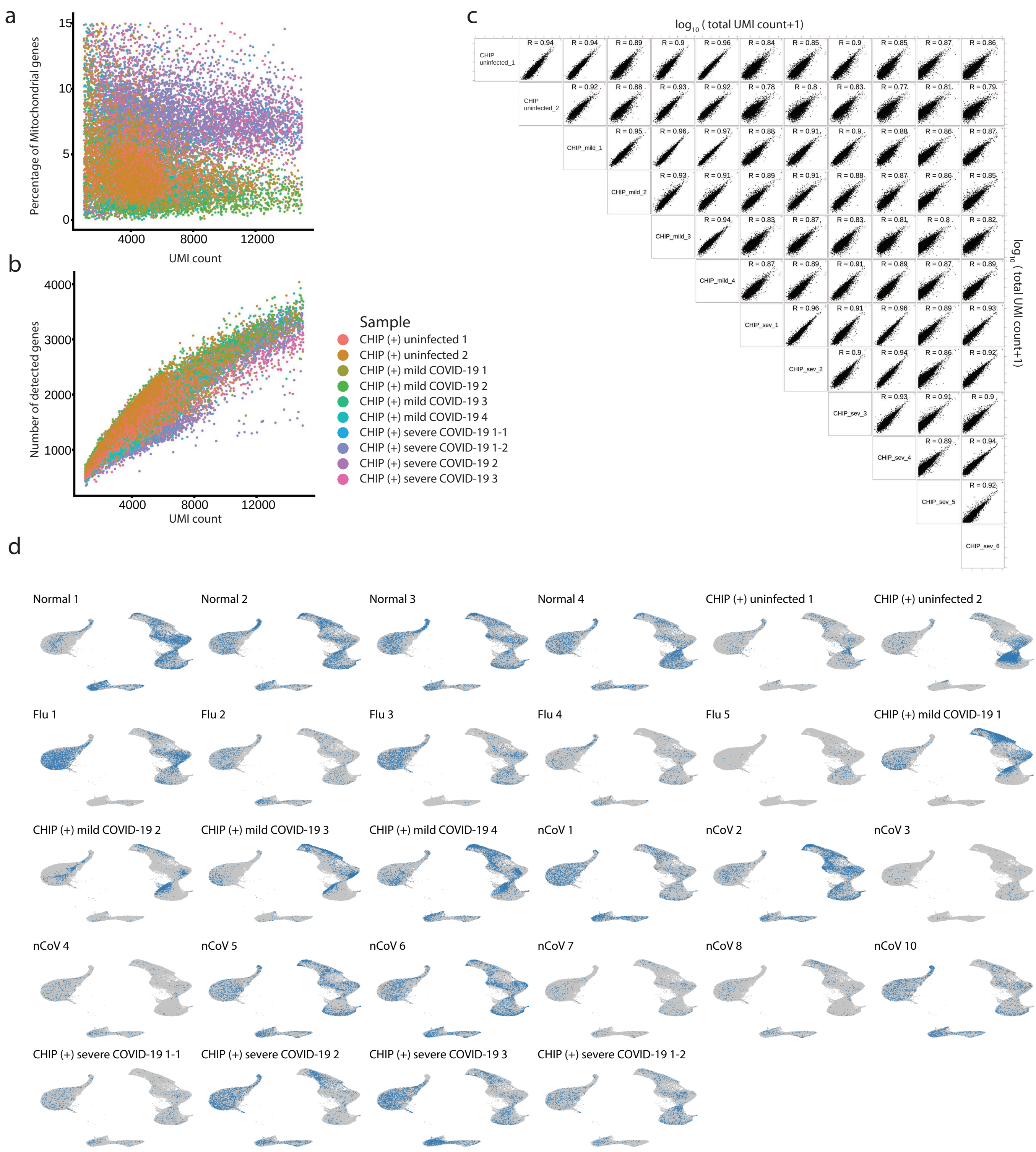

Choi et al Supplementary Fig. 2

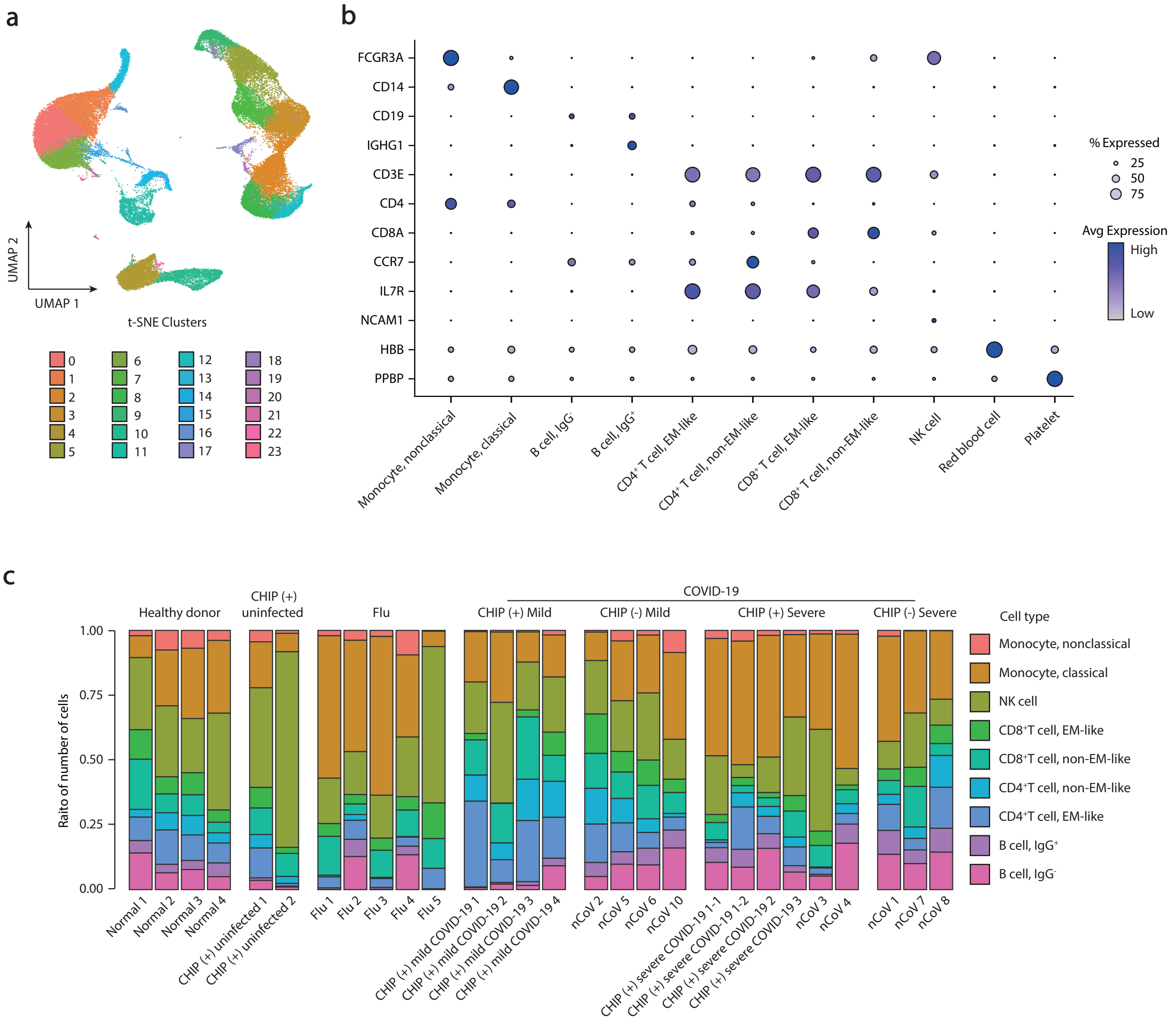

Choi et al Supplementary Fig. 3

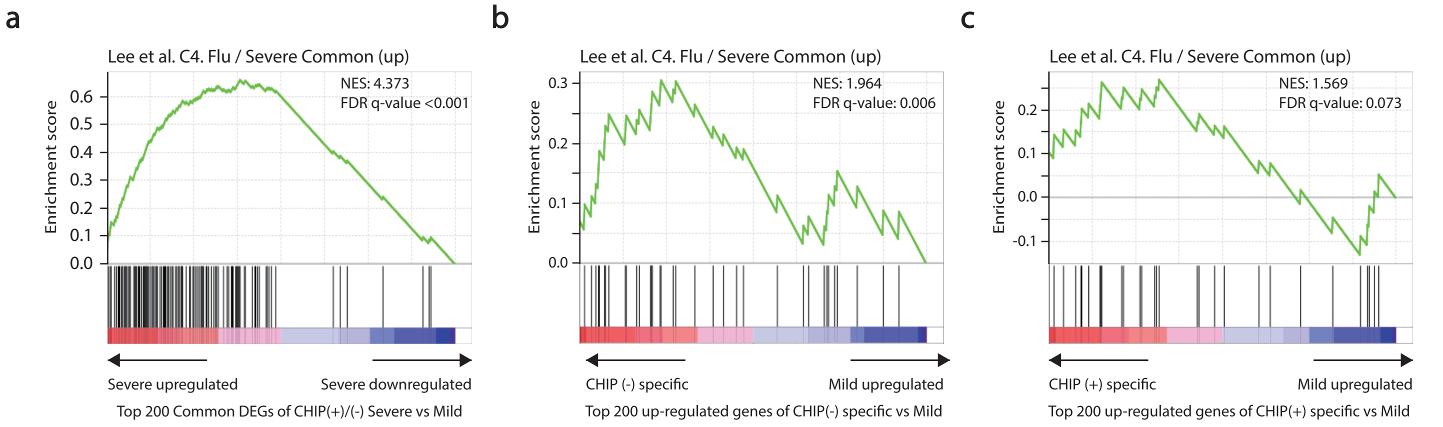

Choi et al Supplementary Fig. 4

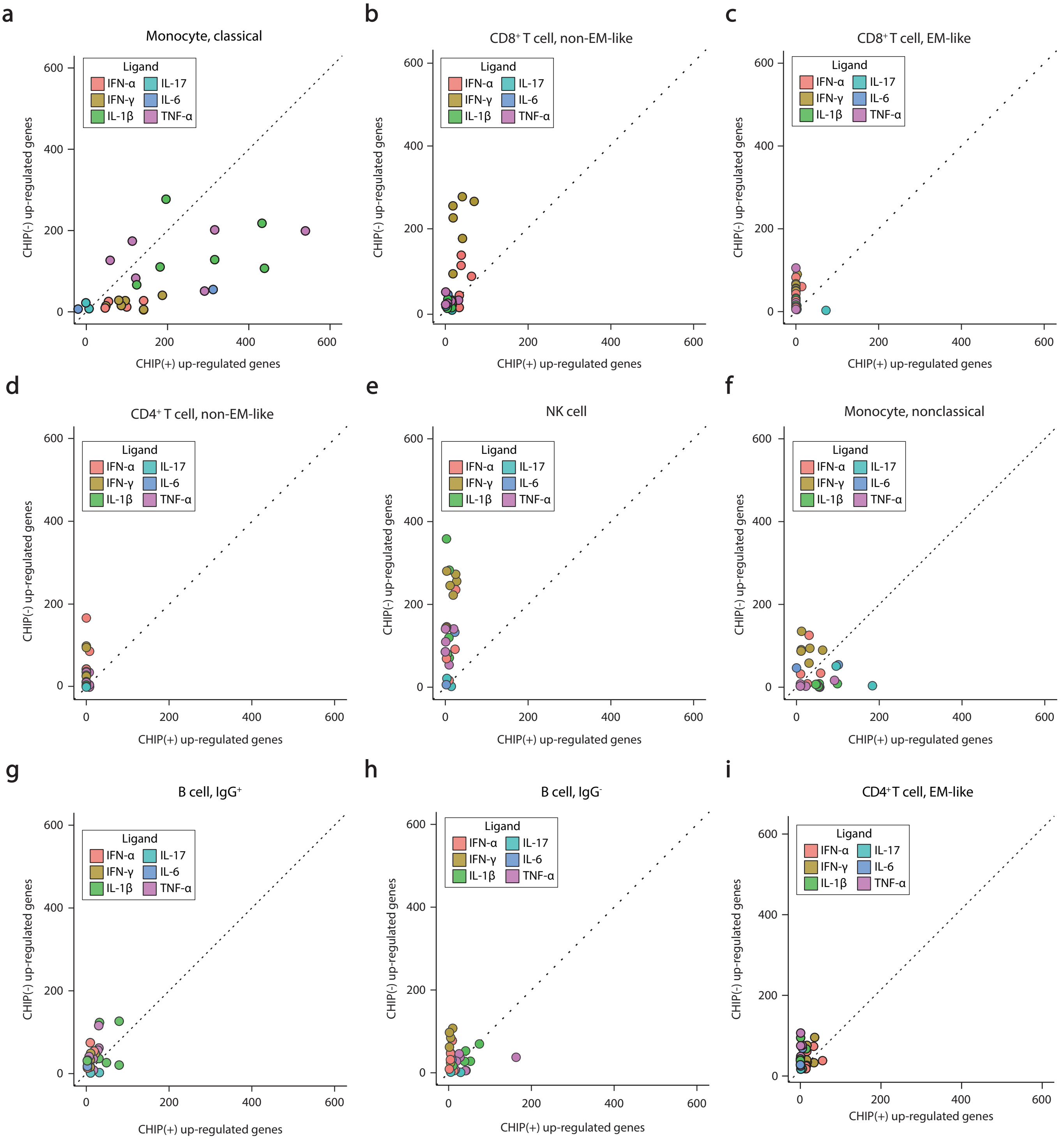

Choi et al Supplementary Fig. 5

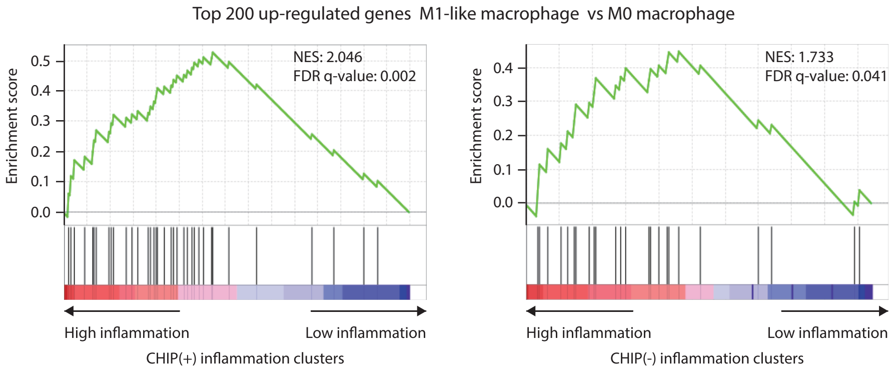

Choi et al Supplementary Fig. 6

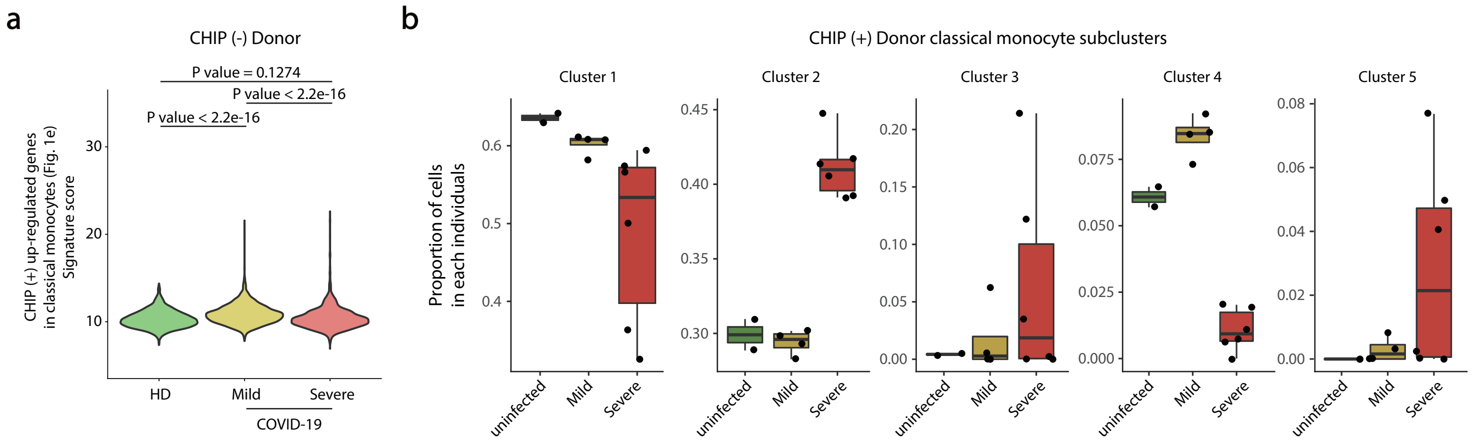

Choi et al Supplementary Fig. 7

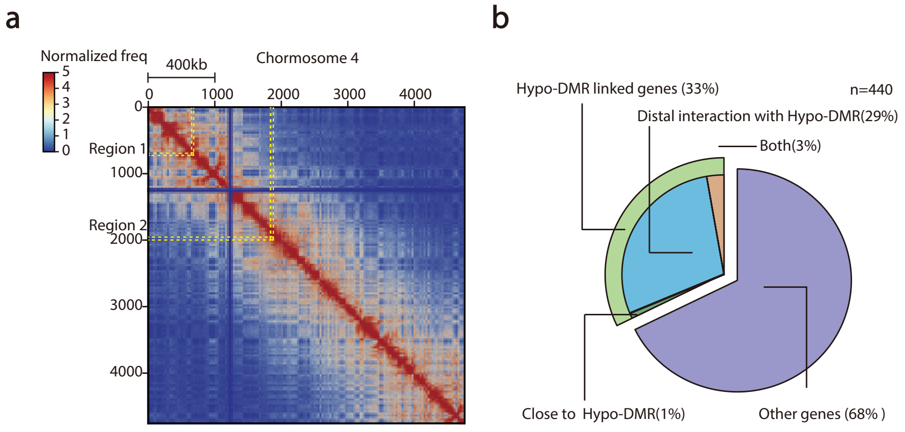

Choi et al Supplementary Fig. 8

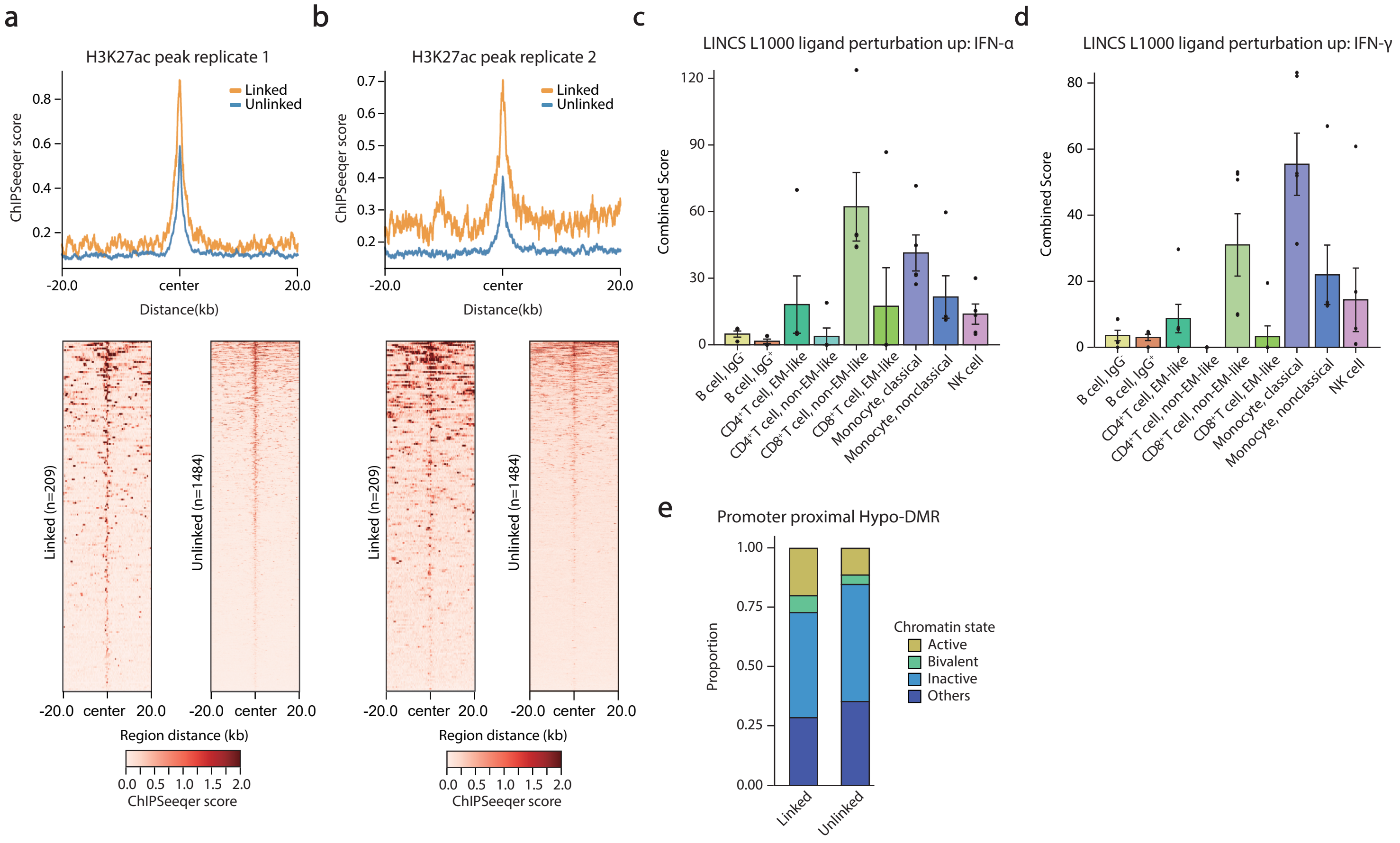

Supplement: Supplementary file 1 — Supplementary Figures, Methods and Legends for supplementary data [file 12276_2022_866_MOESM1_ESM.pdf]
